# The Various Forms of Meningitis

**Published:** 1900-06-09

**Authors:** G. Parker

**Affiliations:** Assistant Physician at the General Hospital, and Lecturer at University College, Bristol.


### Hospital Clinics and Medical Progress.

### THE VARIOUS FORMS OF MENINGITIS.

By G. Parker, M.A., M.D., Assistant Physician at the General Hospital, and Lecturer at University College, Bristol.

The work of separating the different diseases of this group is beset with difficulties, and we have probably not yet reached a final classification. Still less have we determined the causes of each one; yet it is calculated that modern researches have already identified 20 distinct affections of the cerebral meninges; and though a great number of them can be at once dismissed as doubtful varieties, enough remain to afford a wide field for study. About these important varieties very copious observations have been accumulated in the last few years.

An interesting classification is given by C. L. Dana,[1] which it may be useful to take as an outline for discussion. Thus we have:—

I. Pachy-meningitis—(*a*) External: acute or chronic; (*b*) internal: hæmorrhagic, syphilitic, purulent, serous.

II. Lepto-meningitis—(*a*) Simple purulent, (*b*) syphilitic, (*c*) epidemic or cerebro-spinal, (*d*) tubercular, (*e*) posterior basic.

III. Serous meningitis—(*a*) Benign or malignant, (*b*) ventricular or general, (*c*) traumatic, (*d*) chronic.

However, he confesses that only some five or six of these are ordinarily seen, viz.: Internal pachy-meningitis; simple, epidemic, and tubercular lepto-meningitis; and one or more varieties of serous disease. Other writers insist that we must add to them posterior basic. as a well-marked form of distinct origin.

**Pachy-meningitis** has these types. The first group consists of (I. *a*) cases where the dura mater is attacked from without. These are due to injury or disease of the bones. Thus, they are seen after blows or fracture of the skull, disease of the nose or ear, and syphilitic affections of the skull. The symptoms are chiefly those of the surgical disease, to which may be added vertigo, headache, delirium, and convulsions, or the signs of pressure, if pus has accumulated. Of the internal forms (I. *b*) the one usually seen is the syphilitic, but some cases are due to chronic alcoholism, general paralysis, or mal-nutrition, as in scurvy. Among them is the affection called hæmatoma of the dura mater, where membranous layers with the remains of blood between them extend over a large part of the hemispheres. This may be suspected, according to Gowers, where brain failure and headache with apoplectic seizures have occurred in persons who suffer from the effect of injury or chronic degeneration. Very often there are no distinctive symptoms at all. As to the rarer forms of internal pachy-meningitis no special features other than those common to all meningeal diseases can be pointed out.

**Lepto-meningitis.**—The simple suppurative type (II. *a*) is generally a sequel of some septic disease, which has brought about an invasion by strepto- or pneumococci. Thus, pneumonia, empyæma, puerperal, or aural disease often precede it. Anatomically, we find a layer of purulent matter which may cover the anterior part of the upper surface of the brain, though many exceptions occur to this rule. G. F. Still remarks that this disease may often be distinguished from tubercular and other forms by: (1) Its rapid course. The onset is quite sudden, and death usually occurs before the tenth day. (2) The presence of a source of infection such as an empyæma, or disease of the ear. (3) Lumbar puncture, which will show pus cocci or organisms due to the special infection. (4) Vomiting is frequent, the temperature very high, headache severe, and paralysis of cranial nerves is of frequent occurrence. In disease of the ear, however, much difficulty may be felt. For the symptoms of a cerebral abscess resulting from the aural trouble may be easily mistaken for those of meningitis, though the temperature in the former trouble is lower, and the course less rapid. On the other hand, if meningitis is present, it may be tubercular, and not due to the pyogenic organisms in the ear after all.

**Cerebro-spinal Meningitis** (II. *c*) may occur in epidemics or in sporadic cases. In this country few, if any, epidemics have appeared, while on the Continent and in North America there have been a considerable number. The onset is sudden; there is violent headache, vomiting, pain in the limbs, some retraction of the head, marked herpes on the face, while a purpuric rash may attack the legs. We find, too, a high but irregular temperature, and if recovery takes place the disease may be followed by hydrocephalus and various paralyses. The course of the complaint is more rapid than in tubercular meningitis, but less so than in the simple form (II. *a*). The mortality, too, lies between that of these two diseases. C. H. Milnes lays stress on the combination of sudden onset, moderate pyrexia, with slow, full pulse, and nervous symptoms, such as coma or hyperæsthesia. The sporadic cases are, of course, more difficult to diagnose than when we meet with an epidemic, but even here the symptoms form a fairly complete picture, and the herpetic rash sometimes covering both ears is specially noticeable. Again, lumbar puncture will generally show the presence of the diplococcus intracellularis, though in some cases Fränkel's pneumococcus has been found. When the latter is present, though the general symptoms are those of the epidemic disease, it is a little difficult to decide whether we have a case of that or of simple meningitis (II. *a*) to deal with. A marked contrast is seen in

**Tubercular Meningitis** (II. *d*), which is usually slow and insidious in its progress, and is at first frequently mistaken for some kind of gastro-intestinal catarrh, or for typhoid or some other exanthem. However, in patients suffering from advanced tubercular diseases, the onset, as Barlow points out, may be quite sudden. Among early symptoms, after an indefinite feeling of malaise, we find headache, vomiting, retraction of the abdomen, slow, irregular pulse and respiration, the latter being often of the Cheynes-Stokes' character. Optic neuritis of a mild form, and some degree of tremor of the head, together with slight rigidity of the neck, is very common. The sufferers pass through three successive stages of "irritation, pressure, and paralysis" on the average in about three weeks, and nearly always die. The few survivals are probably instances where the disease has attacked a small patch only on the vertex instead of spreading over the whole base, which is the usual mode. The temperature, though high in the latter stages, is raised but little at first, and is markedly irregular. This, too, forms a contrast to the temperature in epidemic or simple purulent meningitis. Lumbar puncture may show Koch's bacillus in the fluid. Paralysis of various cranial nerves are of course often found in this or any disease affecting chiefly the base of the brain, while hydrocephalus is not rare, though the enlargement is usually less than is seen in the basic form, and is due to increased secretion rather than to obstruction.

**Posterior Basic Meningitis** (II. *e*) is a variety described chiefly in young children by Gee, Barlow, and Lees, and remarkable for the extreme retraction of the head seen in it, while the mortality is comparatively low. The infecting organism here is said by Still to be a special diplococcus, nearly resembling that of epidemic meningitis, but differing in its longer vitality and a few other respects. The course of the disease is slow, lasting on the average seven weeks. There is persistent vomiting, and the head retraction is often so extreme that the occiput comes near the spine or even the buttocks. The onset is sudden, sensation remains normal, and the pyrexia is irregular. Hydrocephalus from obstruction is wont to supervene in all but the mildest cases, and tonic spasm of the limbs may accompany the retraction of the head. A considerable number of cases recover, but the hydrocephalus is apt to be permanent.

**Syphilitic Lepto-meningitis** (II. *b*) is exceedingly chronic, and, according to Gowers, is generally associated with choroiditis and with gummata or vascular changes which cause persistent paralysis of one or more cranial nerves. The affection is very often local, extending only over a small part of the pia mater, with few general symptoms and perhaps only a single cranial nerve affected. Occasionally it would seem to result in a state not unlike that of "general paralysis," but there is not much tremor, and rarely grandiose ideas, and usually the onset is apoplectiform. Nocturnal headache will sometimes give a clue to the cause of such meningitis.

**Serous Meningitis** is really a form of hydrocephalus, as a watery and not a purulent secretion is present. Thus there is an acute (III. *c*) traumatic œdema which follows blows on the head. The symptoms last about three days, and include slight rigidity of the neck, pains in the head, pyrexia, and contracted pupils, followed usually by recovery. We may notice, too, an acute œdema (III. *a*) in alcoholics, insane persons, and those suffering from starvation or certain narcotics. This is known in asylums as "wet brain," and lasts about ten days. There is some delirium, moderate pyrexia, and rigidity of neck, but no convulsions, and the fluid obtained by lumbar puncture is sterile. The symptoms differ from those in suppurative meningitis in that there is less fever and headache, no optic neuritis, and the onset is more gradual. There may also be hyperæsthesia and some muscular twitchings (Dana). The type described by Quincke is also an acute one, and seen in children or young persons. It is probably due to an infection, but no organism has yet been identified from it. In some instances it proves quickly fatal, while other patients have recovered after a short illness. Permanent hydrocephalus may result, and relapses sometimes occur. In only a few cases is there any pyrexia, but headache, delirium, coma, and slight rigidity of the neck seem to be usual. We have not space for a discussion of other forms of meningitis, but those usually seen belong to one or other of the types we have discussed above.

[1] Journal of Nervous and Mental Diseases, Dec.

